# RNA Sequencing Analysis of Chicken Cecum Tissues Following *Eimeria tenella* Infection in Vivo

**DOI:** 10.3390/genes10060420

**Published:** 2019-05-31

**Authors:** Xiaohui Wang, Wenbin Zou, Hailiang Yu, Yuxin Lin, Guojun Dai, Tao Zhang, Genxi Zhang, Kaizhou Xie, Jinyu Wang, Huiqiang Shi

**Affiliations:** 1 College of Animal Science and Technology, Yangzhou University, Yangzhou 225009, Jiangsu, China; wxh9409161412@126.com (X.W.); wenbinzou1216@163.com (W.Z.); hailiangyu122514@163.com (H.Y.); yu_xin_lin@163.com (Y.L.); zhangt@yzu.edu.cn (T.Z.); zgx1588@126.com (G.Z.); yzxkz168@163.com (K.X.); jywang@yzu.edu.cn (J.W.); 2 Animal Husbandry and Veterinary Station of Kunshan City, Kunshan 215300, Jiangsu, China; 3 Jiangsu Jinghai Poultry Group Co., Ltd., Haimen 226100, Jiangsu, China; 13921648510@163.com

**Keywords:** RNA-seq, *E. tenella*, chicken, cecum, differentially expressed genes

## Abstract

*Eimeria tenella* (*E. tenella*) is one of the most frequent and pathogenic species of protozoan parasites of the genus *Eimeria* that exclusively occupies the cecum, exerting a high economic impact on the poultry industry. To investigate differentially expressed genes (DEGs) in the cecal tissue of Jinghai yellow chickens infected with *E. tenella*, the molecular response process, and the immune response mechanism during coccidial infection, RNA-seq was used to analyze the cecal tissues of an *E. tenella* infection group (JS) and an uninfected group (JC) on the seventh day post-infection. The DEGs were screened by functional and pathway enrichment analyses. The results indicated that there were 5477 DEGs (*p*-value < 0.05) between the JS and the JC groups, of which 2942 were upregulated, and 2535 were downregulated. GO analysis indicated that the top 30 significantly enriched GO terms mainly involved signal transduction, angiogenesis, inflammatory response, and blood vessel development. KEGG analysis revealed that the top significantly enriched signaling pathways included focal adhesion, extracellular matrix–receptor interaction, and peroxisome proliferator-activated receptor. The key DEGs in these pathways included *ANGPTL4, ACSL5, VEGFC, MAPK10*, and *CD44*. These genes play an important role in the infection of *E. tenella*. This study further enhances our understanding of the molecular mechanism of *E. tenella* infection in chickens.

## 1. Introduction

Avian coccidiosis, caused by seven species of protozoan parasites of the genus *Eimeria*, is one of the most important livestock diseases in the world [1], resulting in high economic impacts by reducing animal performance and lowering productivity [2]. So far, coccidiostats and live vaccines are the two major coccidiosis control strategies [3]. However, extensive use of coccidiostats has resulted in drug resistance and raised public concern for food safety because of its chemical residues in food-producing animals [4]. The use of live vaccines has the risk of causing reversion to a pathogenic strain [5]. Although DNA vaccines that comprise genes encoding immunogenic proteins of pathogens have been investigated as vaccine candidates, the price of any new vaccine and the genetic background of the recipient will continue to receive major consideration in the global poultry industry [6]. Genetic selection of coccidiosis-resistant lines by novel molecular genetics and functional genomics tools provides the potential to radically address this problem [7]. However, our understanding of how chickens respond to coccidian infection at the molecular level is limited [8].

RNA-seq is an approach to transcriptome profiling that provides a far more precise measurement of the levels of transcripts and their isoforms than other methods [9]. Several studies used transcriptome gene chip hybridization of intraepithelial lymphocytes or ceca from infected and noninfected chickens to study gene expression profile changes in response to *Eimeria tenella* at 0–6 days post-infection (PI) [8,10]. Scanning electron microscopy showed that coccidioides invaded mainly the cecum of chickens, damaged the intestinal mucosa, and caused cecal swelling, bleeding, and excretion of large amounts of bloody stool on the fourth day after infection; from 8 to 10 days PI, the damaged mucosal surface became normal [11,12]. Guo et al. [8] performed significance analysis of microarray (SAM) of chicken cecum mucosa after 4.5 days of *E. tenella* infection using a gene chip to explore the molecular mechanisms of coccidiosis infection. In this study, we applied the RNA-seq technique to screen the differentially expressed genes (DEGs) between *E. tenella*-infected and uninfected cecal tissues of Jinghai yellow chickens on the seventh day PI and analyze the GO terms and pathways of these DEGs, so to find the main DEGs associated with *E. tenella* infection and to provide a reference for breeding new varieties or new strains with coccidiosis resistance.

## 2. Materials and Methods

### 2.1. E. tenella Infection in Chickens and Tissue Collection

*E. tenella* was originally isolated in the field in Yangzhou, China, and maintained in the Department of Parasitology, College of Veterinary Medicine, Yangzhou University, Jiangsu Province, China. Parasite oocysts were harvested, sporulated, and stored as previously described [13]. The experimental animal was the Jinghai yellow chicken, characterized by small size, good meat quality, disease and stress tolerance compared to other chickens of the same type. A total of 12 female, half siblings chicks with similar body weights were randomly selected (purchased from Jinghai Yellow Chicken Resource Farm, Haimen, Jiangsu Province, China), housed in oocyst-free cages, and fed antibiotic-free feed for up to 30 days. All chickens were determined to be free from parasitic infection after fecal detection, were randomly divided into an infected group (JS) and an uninfected group (JC), and were kept in single cages, maintained free of coccidioides by flame disinfection with a gasoline torch. Each chicken in the infected group was subjected to oral infection with 2.5 × 10^4^ *E. tenella* sporulated oocysts. The uninfected group received the same amount of normal saline. On the seventh day PI, the cecum tissues of the three chickens with the highest cecal lesion scores [14] in the infected group (denoted JS1-JS3) and the cecum tissues of three chickens randomly selected from the uninfected group (denoted JC1-JC3) were collected for total RNA extraction. All animal protocols were approved by the Animal Welfare Committee of Yangzhou University, and all efforts were made to minimize the suffering of the chickens.

### 2.2. RNA Isolation and Quality Assessment

Total RNA from six cecum samples was extracted using TRIzol reagent (Invitrogen, Carlsbad, CA, USA) according to the manufacturer’s instructions. RNA degradation was monitored in 1% agarose gels. RNA purity was checked using a NanoPhotometer^®^ spectrophotometer (IMPLEN, MD, CA, USA). RNA concentration was measured using a Qubit^®^ RNA Assay Kit with a Qubit^®^ 2.0 Fluorometer (Life Technologies, South San Francisco, CA, USA). RNA integrity was assessed using an RNA Nano 6000 Assay Kit with a Bioanalyzer 2100 system (Agilent Technologies, Santa Clara, CA, USA).

### 2.3. Library Preparation and Transcriptome Sequencing

Qualified total RNA for each of the six cecum tissues was sent to Novogene Biological Information Science and Technology Company (dry ice preservation) for library construction and transcriptome sequencing. Sequencing libraries were generated using an NEBNext^®^ Ultra™ RNA Library Prep Kit for Illumina^®^ (NEB, Ispawich, CA, USA) according to the manufacturer’s recommendations, and index codes were added to attribute sequences to each sample. PCR products were purified (AMPure XP system) (Beckmankurt life sciences division, Indianapolis, Indiana, USA), and library quality was assessed on an Agilent Bioanalyzer 2100 system.

Clustering of the index-coded samples was performed on a cBot Cluster Generation System using a TruSeq PE Cluster Kit v3-cBot-HS (Illumina) according to the manufacturer’s instructions. After cluster generation, the library preparations were sequenced on an Illumina HiSeq 2000 platform, and 100 bp paired-end reads were generated. The raw data were uploaded to the NCBI Sequence Read Archive (SRA); the accessions in the SRA for the submission is SRP198721.

### 2.4. Sequence Read Mapping to the Gallus Gallus Reference Genome

Raw data (raw reads) in fastq format were first processed through in-house Perl scripts. In this step, clean data (clean reads) were obtained by removing reads containing adapter sequences, reads containing poly-N, and low-quality reads from the raw data. Furthermore, quality parameters of Q20, GC content, and sequence duplication level were used for data filtering. All the subsequent analyses were based on the high-quality clean data.

Reference genome and gene model annotation files were downloaded from the genome website (ftp://ftp.ensembl.org/pub/release-75/fasta/gallus_gallus/dna/). The index of the reference genome was built using Bowtie v2.0.6 (Johns Hopkins University, Baltimore, MD, USA) [15], and paired-end clean reads were aligned to the reference genome using TopHat v2.0.9 (Johns Hopkins University, Baltimore, MD, USA) [16]. TopHat was selected as the mapping tool because it can obtain a better mapping result than other nonsplice mapping tools. The Cufflinks v2.1.1 reference annotation-based transcript (RABT) (Johns Hopkins University, Baltimore, MD, USA) assembly method was used to construct and identify both known and novel transcripts from TopHat alignment results [17].

### 2.5. Quantification and Differential Expression Analysis of Transcripts

HTSeq v0.5.4p3 (Stanford University, CA, USA; Heidelberg University, Baden-Württemberg, Germany) was used to count the read numbers mapped to each gene [18]. In addition, the RPKM (reads per kilobase of exon model per million mapped reads) of each gene was calculated based on the length of the gene, and read counts were mapped to it. RPKM considers the effect of sequencing depth and gene length for the read counts at the same time and is currently the most commonly used method for estimating gene expression levels [19]. Differential expression analysis of the two groups (three biological replicates per condition) was performed using the DESeq package [20]. The resulting *p*-values were adjusted using Benjamini and Hochberg’s [21] approach for controlling the false discovery rate. Genes with an adjusted *p*-value < 0.05 found by DESeq were identified as differentially expressed.

### 2.6. GO and KEGG Enrichment Analysis of Differentially Expressed Genes

Gene ontology (GO) enrichment analysis of DEGs was implemented by the GOseq R package (the University of Auckland, Auckland, New Zealand) [22], in which gene length bias was corrected. GO terms with corrected *p*-values less than 0.05 were considered significantly enriched for DEGs.

KEGG is a database resource for understanding high-level functions and utilities of the biological system, such as the cell, the organism, and the ecosystem, from molecular-level information, especially large-scale molecular datasets generated by genome sequencing and other high-throughput experimental technologies (http://www.genome.jp/kegg/). We used KOBAS software [23,24] to test the statistical enrichment of DEGs in KEGG pathways.

### 2.7. Validation of Differentially Expressed Genes by qRT-PCR

Ten DEGs were randomly selected. The primers used for quantification in the study were designed using Primer-BLAST on the NCBI website. In all cases, primers designed for qRT-PCR spanned exon–exon boundaries. Primer sequences can be seen in Table 1. *YWHAZ* and *TBP* were used as reference genes [25]. qRT-PCR was performed using an ABI Prism 7500 sequence-detection system (Applied Biosystems, Foster City, CA, USA) with SYBR Green PCR Master Mix (TaKaRa, Dalian, China), according to the manufacturer’s instructions. Relative expression was calculated using the delta-delta-Ct method. The qPCR reaction system was: 2×ChamQ SYBR qPCR Master Mix 10 μL; forward and reverse primers, 0.4 μL each; 50× ROX Reference Dye 0.4 μL; 2 μL template; addition of 6.8 μL ddH_2_O to 20 μL. qPCR response procedures: 40 cycles at 95 °C for 30 s, 95 °C for 10 s, 60 °C for 30 s.

## 3. Results

### 3.1. Clinical Observation

On days 1–3 post-infection, the chickens in each group had no special symptoms. On the fourth day post-infection, the infected group began to excrete bloody diarrheas, and especially on the fifth day after infection, bloody diarrheas excretion was the highest. On the sixth day post-infection, bloody diarrheas excretion began to decrease. The infected chickens appeared listlessness, wasted food, and presented anal filth and paralytic and spasm symptoms. No chicken deaths occurred in the infected group during the trial period. On the eighth day, the infected chickens were found to have some cecum swelling and blackening. The chickens in the control group showed normal behavior, and their growth state was not different from that of normally fed chickens.

### 3.2. RNA-Seq Data Analysis

Transcriptome sequencing results and quality parameters are shown in Table 2. The number of raw reads per sample that were 100 bp in length ranged from 52,432,520 to 64,910,184, indicating high abundance. The GC content reached between 49.18% and 50.62%. This result indicated that the GC content of each sample was relatively consistent. The percentage of Q20 bases in each sample was greater than 96%. To remove the interference of the adapters and low-quality reads during transcriptional information analysis, the six samples were filtered for reads with adapters, low quality, and a ratio of N (N indicates that the bases could not be determined) greater than 10%. The total clean read length was 34.8 gigabases (Gb). Approximately 80% of the total reads mapped to the chicken chromosomes, and over 78% of the reads mapped uniquely. These results further confirmed the reliability of the RNA-seq analysis and the sampling accuracy of the cecum tissue used in this study.

### 3.3. Identification of Differentially Expressed Genes in Chicken Ceca upon E. tenella Infection

**Figure 1:**
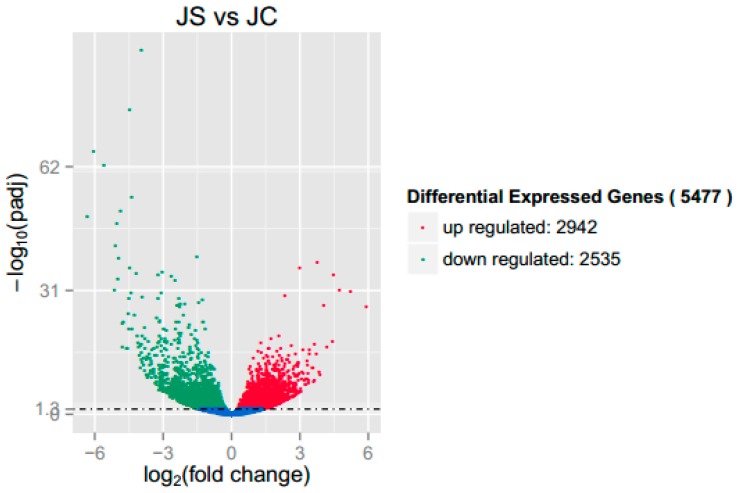
Volcano plot of differentially expressed genes (DEGs) between the infected group (JS) and the uninfected group (JC). Note: The red dots and green dots indicate significantly upregulated and downregulated genes, respectively (padj < 0.05). The blue dots represent the genes whose difference in expression level between the JS and the JC groups did not reach significance (padj > 0.05).

In this study, fold change and *p*-value were evaluated and screened for DEGs. A fold change of more than 2 and a significance level of padj < 0.05 were considered to be significant. Using a volcano plot (Figure 1), we could see that 5477 DEGs were significantly changed, including 2942 upregulated genes and 2535 downregulated genes.

### 3.4. GO Enrichment and KEGG Pathway Analysis for DEGs

**Figure 2:**
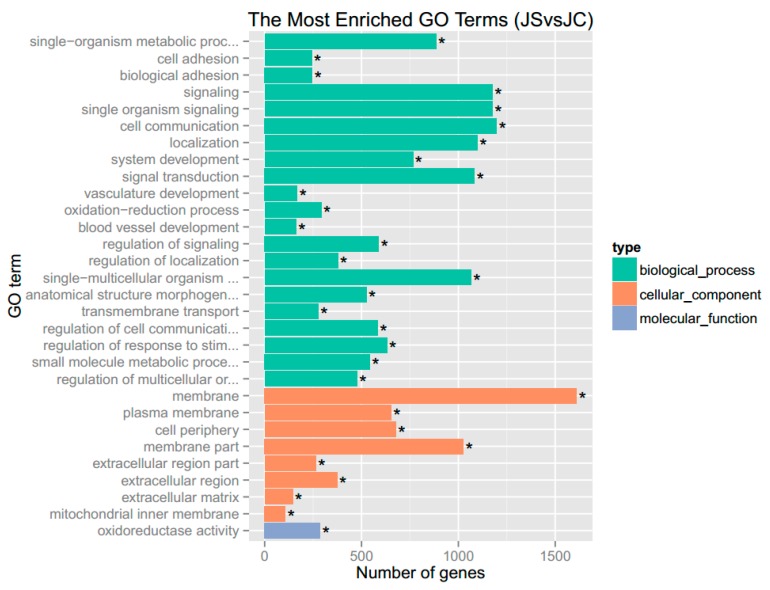
The top 30 significantly enriched gene ontology (GO) terms. Note: “*” indicates the significance (padj < 0.05) of enriched GO terms.

GO analysis showed that there were 11236 GO terms enriched with DEGs, of which 545 were significantly enriched, including terms related to biological process, cellular component, and molecular function. The top 30 significantly enriched GO terms are shown in Figure 2. Biological process analysis showed that the largest groupings of DEGs were related to cell communication (GO:0007154), signaling (GO:0023052), single-organism signaling (GO:0044700), signal transduction (GO:0007165), and single-multicellular organism process (GO:0044707). Similarly, molecular function analysis of these genes revealed that the most prominent function was related to oxidoreductase activity (GO:0016491).

**Figure 3:**
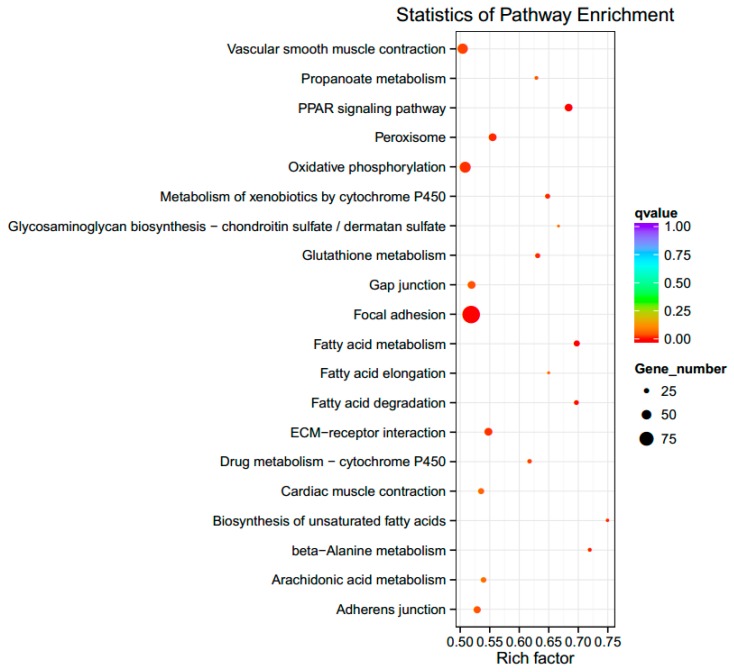
The top 20 significantly enriched functional KEGG pathways. Note: The size of the dot represents the number of differentially expressed genes in the pathway. The color of the dot corresponds to different ranges of *q*-value (corrected *p*-value < 0.05). The vertical axis represents the enriched pathways. Rich factor refers to the ratio of the number of differential genes enriched to the number of annotated genes enriched in the pathway. The higher the rich factor value, the greater the enrichment degree will be. PPAR: peroxisome proliferator-activated receptor, ECM: extracellular matrix.

The KEGG pathways of the DEGs are shown in Figure 3. The figure shows the top 20 pathways with the smallest *q*-value. Among them, the three most significantly enriched pathways were peroxisome proliferator-activated receptor (PPAR) signaling, focal adhesion, and extracellular matrix (ECM)–receptor interaction, for which there were 24, 55, and 24 enriched DEGs, respectively. The genes in these three pathways that are involved in the immune response were *ANGPTL4, ACSL5, VEGFC, MAPK10¡,* and *CD44*. Relevant information is shown in Table 3.

### 3.5. Real-Time PCR Validation of Differential Gene Expression in Chicken Ceca

Among the DEGs, five upregulated and five downregulated DEGs were randomly selected to verify the reliability of the RNA-seq results by quantitative real-time PCR (qRT-PCR). The results demonstrated that the fold change of the upregulation and downregulation of the 10 selected DEGs was basically the same using two different detection methods, and the correlation coefficient of the two reached 0.988 (R^2^ = 0.975), which was highly significant (*p* < 0.000). The results from qRT-PCR analysis confirmed the high reproducibility of the RNA-seq data in this study (Table 4).

## 4. Discussion

Coccidiosis of chickens is an acute parasitic disease that seriously damages the breeding industry and causes great economic loss to the commercial poultry industry [10]. Traditional methods for the prevention of coccidiosis have many disadvantages. Therefore, functional genomics and bioinformatics have become powerful techniques for studying host–pathogen interactions in coccidiosis. Studies have identified new candidate genes that influence the host immune responses to *Eimeria* spp. using chicken macrophage and lymphocyte cDNA microarrays [26]. Kim et al. [10] used a cDNA microarray to study gene expression in isolated intestinal lymphocytes and found a large number of genes significantly regulated by coccidial infection. Guo et al. [8] performed SAM of chicken cecum mucosa after 4.5 days of *E. tenella* infection using gene chip to explore the molecular mechanisms of coccidiosis infection and identified 7099 DEGs (4033 upregulated and 3066 downregulated). GO and KEGG analysis showed that the upregulated genes were mainly involved in immunity and defense, apoptosis and cell death, differentiation, signal transduction, and extracellular matrix (ECM) composition, while the downregulated genes mainly related to the membrane components and some transporters. In our study, the transcriptome library of the cecum tissues of chickens in the *E. tenella* JS and JC group was analyzed. A total of 5477 DEGs were detected. Among them, 2942 genes were upregulated, and 2535 genes were downregulated. The key DEGs linked to coccidial infection included *ANGPTL4, ACSL5, VEGFC, MAPK10, CD44*. KEGG pathway enrichment results showed that the main pathways related to these DEGs were PPAR signaling, focal adhesion, and ECM–receptor interaction. The comparison between this study and Guo et al. study showed that the sampling time was different, the number of DEGs was not the same, and the GO terms and majority of KEGG pathways related the identified DEGs were also different. Only the EMC–receptor interaction pathway was significantly enriched in both this study and the study by Guo et al.

PPARs are cell surface receptors that act as transcription factors and belong to the ligand-activated nuclear receptor superfamily [27]. In addition to mediating anti-inflammatory effects, PPARs participate in cell proliferation and differentiation, angiogenesis, apoptosis, and immune and inflammatory responses. Studies have shown that PPARs have a variety of inhibitory effects on inflammation, including reducing the transcriptional activity of NF-kB and the production of proinflammatory molecules in T lymphocytes, promoting the expression of anti-inflammatory mediators in the innate immune system, and inhibiting the genes encoding proinflammatory molecules in macrophages [28]. Focal adhesions are important cytoskeletal structures that not only maintain the normal structure of a cell but also participate in the normal functions of cell migration, movement, gene expression, survival, and apoptosis [29]. Because of the widespread tissue destruction caused by *Eimeria* protozoan invasion, it is tempting to hypothesize that part of the host’s physiological response in the gut to infection involves tightening of the cecal epithelial barrier, which may be accomplished in part by strengthening the interaction between epithelial cells and the extracellular matrix through focal adhesions [30]. As cell surface receptors, ECM receptors can specifically bind to other cell surface receptors, activate signal cascades, regulate neuron functions, and play an important role in controlling key events such as cell adhesion, migration, proliferation, differentiation, and survival [31]. Zhang et al. [31] performed RNA sequencing and KEGG pathway analysis of patients with hepatocellular carcinoma and found nine enriched pathways, among which were ECM–receptor interaction, focal adhesion, and TGF-β signaling. Guo et al. [8] found through transcriptome analysis, that the upregulated genes were mainly associated with immunity and defense (e.g., natural killer cell-mediated cytotoxicity) and ECM (e.g., ECM–receptor interaction) after infection of chicken cecum epithelial cells with *E. tenella.* This finding is consistent with the results of this experiment. Therefore, these significantly enriched pathways play a regulatory role in the process of host infection by coccidioides.

Angiopoietin-like 4 (ANGPTL4) is an important regulatory factor that can modulate vascular permeability and reactive oxygen species levels and promote angiogenesis and wound healing [32]. Studies have shown that ANGPTL4 stimulates endothelial cell growth and differentiation. More importantly, the combination of VEGF and ANGPTL4 protects endothelial cells from inhibition by rosiglitazone [33]. *ACSL5* has been associated with cell development and maturation, physiopathological processes, apoptosis, and tumorigenesis [34]. Several in vivo and in vitro studies have indicated that *ACSL5* may play an important role in the immune dysfunction of lupus-like mouse models [35]. In this study, *E. tenella* infection led to intestinal mucosal damage and caecal bleeding; the *ANGPTL4* gene and the *ACSL5* gene can promote cell development and angiogenesis, and they were both significantly upregulated in the PPAR pathway on the seventh day after chicken infection with coccidioides. Therefore, these two genes may be involved in the process of host response to coccidial infection.

CD44 is a cell surface adhesion receptor that regulates metastasis via recruitment of CD44 to the cell surface. Its interaction with appropriate ECM ligands can promote migration and invasion processes in the process of cell metastasis [36]. DeGrendele et al. [37] showed that a combination of T cells and antibodies can induce the generation of CD44-HA and promote the infiltration of lymphocytes in inflammatory sites. Our study found that *CD44* was significantly expressed in the the ECM–receptor interaction pathway.

We also observed that multiple DEGs associated with the significantly enriched focal adhesion pathway were either up- or downregulated during *E. tenella* infection. Vascular endothelial growth factor C (VEGFC) is a vascular epithelial growth factor. Its main function is to affect lymphatic epithelial cells together with its receptor VEGFR-3 (FLT4), thereby promoting cell growth and proliferation and regulating the growth of lymphatic vessels [38]. It has been reported that the interaction between VEGFC and VEGFR-3 mediates lymphatic endothelial cell (LEC) proliferation, survival, migration, and tube formation during the lymphangiogenesis process [39]. MAPK10, a member of the Jun N-terminal kinase subgroup of mitogen-activated protein kinases, has proapoptotic functions and can function as a tumor-suppressor protein to suppress tumorigenesis [40]. In this study, DEGs were significantly enriched in the focal adhesion pathway, and their expression levels were significantly higher than in the control group. The results showed that these DEGs were involved in the immune response, apoptosis, and angiogenesis processes with *E. tenella* infection.

In conclusion, this study systematically reveals the DEGs and significantly enriched GO terms and KEGG pathways between an *E. tenella*-infected group and an uninfected group using RNA-seq technology. We found that the signaling pathways with the most significant enrichment of differentially expressed genes between infected group and uninfected group included focal adhesion, extracellular matrix–receptor interaction, and peroxisome proliferator-activated receptor, which may play a role in the chicken’s response to *E. tenella* infection. The results further enhance our understanding of the molecular response process and immune response mechanism during *E. tenella* infection.

## Figures and Tables

**Table 1:** Primers used for the qRT-PCR assays.

Gene	Forward primers (5′-3′)	Reverse primers (5′-3′)	Length (bp)	GenBank ID
*ANPEP*	GCCCACCTGGGACATTAAAGA	GATCTGTGCTGGGGTGTTGA	127	NM_204861.1
*HKDC1*	AAAACCCACCCCCAATACCC	CCAGGGTTAGCACGAAAGGT	193	XM_421579.4
*CA7*	GAGGCCGCCAATGGAAGAG	CACTCCGAAGGTCCCGC	102	XM_004944247.1
*BEST4*	GATTCCCTGCGTCTGGTTCA	AATGGTAACCACCTGCGTGT	181	NM_001252125.1
*OTOP2*	GTGGCTCCTGAAAGTGGGAA	CCCCACGTTCTTCCACATGA	166	XM_003642368.2
*COL8A1*	CAGTTGTTTCGCACCCAAGG	ACTCTGTTCTCAGTCGCTGT	187	NM_001293134.1
*TAC1*	CGTCCGTCCATCAGTGTGTT	CAGCTCCTCCTTCTGCTCG	180	XM_004939318.1
*CHRDL2*	TGAACCCAAAACGGCCAGAT	TAGCACCTCGTGTTGCCATT	145	XM_004939009.1
*CEBPB*	CTCCTACCTGGGCTACCAGT	TTGTACTCGTCGCTGTGCTT	195	NM_205253.2
*CTSL2*	AAAGACCAGGGTCAGTGTGG	TTGATTTCCTTCTGGGCGGG	141	NM_001168009.1
*YWHAZ*	GTTCCCTTGCAAAAACGGCTT	AGACGGAAGTTGGAAGGCTG	194	NM_001031343.1
*TBP*	GAACCACACCTCTGTACCCG	GCAGCAAAACGCTTGGGATT	196	NM_205103.1

**Table 2:** Summary of sequencing reads mapping to the reference genome and quality parameters.

Sample	Raw Reads	Clean Reads	Total Mapped	Uniquely Mapped	Q20 (%)	GC (%)
JS1	64,910,186	60,434,754	48,799,256	48,250,954	96.01	49.18
JS2	52,432,520	49,658,574	39,706,701	39,238,872	96.49	50.16
JS3	61,872,638	58,613,210	46,465,172	45,966,122	96.52	50.60
JC1	63,030,840	60,429,518	49,847,352	48,938,857	97.18	50.62
JC2	60,667,018	57,459,296	47,046,823	46,281,102	96.67	50.25
JC3	64,190,770	61,549,834	51,224,793	50,430,317	97.26	49.75

**Table 3:** KEGG pathway analysis of DEGs.

Pathway Name	Pathway ID	DEG Number	*q*-value
PPAR signaling	gga03320	24	0.003765846
Focal adhesion	gga04510	55	0.009483415
ECM–receptor interaction	gga04512	24	0.079280157

**Table 4:** Validation of RNA-seq data by qRT-PCR.

Gene	Gene ID	RNA-seq log_2_(Fold Change)	qRT-PCR log_2_(Fold Change)
*OTOP2*	ENSGALG00000007791	−6.3318	−6.1629
*CA7*	ENSGALG00000005178	−5.6135	−5.4033
*HKDC1*	ENSGALG00000021039	−4.4784	−3.8074
*BEST4*	ENSGALG00000010126	−4.3859	−3.9561
*ANPEP*	ENSGALG00000027501	−3.9646	−3.7506
*CTSL2*	ENSGALG00000012610	+2.0931	+2.2265
*CEBPB*	ENSGALG00000008014	+2.3416	+3.2373
*TAC1*	ENSGALG00000009737	+3.0046	+4.0644
*COL8A1*	ENSGALG00000015253	+3.7752	+4.4074
*CHRDL2*	ENSGALG00000017308	+5.2340	+3.7442

Note: “+” indicates upregulated genes, and “−” indicates downregulated genes.
